# Inhibition of placental mTOR signaling provides a link between placental malaria and reduced birthweight

**DOI:** 10.1186/s12916-016-0759-3

**Published:** 2017-01-03

**Authors:** Kris Genelyn Dimasuay, Elizabeth H. Aitken, Fredrick Rosario, Madi Njie, Jocelyn Glazier, Stephen J. Rogerson, Freya J. I. Fowkes, James G. Beeson, Theresa Powell, Thomas Jansson, Philippe Boeuf

**Affiliations:** 1 Department of Medicine at Royal Melbourne Hospital, The University of Melbourne, Parkville, 3004 VIC Australia; 2 Centre for Biomedical Research, Burnet Institute, 85 Commercial Road, Melbourne, 3004 VIC Australia; 3 Department of Obstetrics & Gynecology, University of Colorado Anschutz Medical Campus, Aurora, CO USA; 4 Maternal and Fetal Health Research Centre, Division of Developmental Biology & Medicine, School of Medical Sciences, Faculty of Biology, Medicine & Health, University of Manchester, St. Mary’s Hospital, Manchester, UK; 5 Victorian Infectious Diseases Service, Royal Melbourne Hospital, Parkville, VIC Australia; 6 Centre for Epidemiology and Biostatistics, Melbourne School of Population and Global Health, The University of Melbourne, Melbourne, VIC Australia; 7 Department of Epidemiology and Preventive Medicine, Department of Infectious Diseases, Monash University, Melbourne, VIC Australia; 8 Department of Microbiology and Central Clinical School, Monash University, Clayton, 3800 VIC Australia; 9 Department of Pediatrics, University of Colorado Anschutz Medical Campus, Aurora, CO USA

**Keywords:** Intervillositis, Fetal growth restriction, System A transporter, Deptor

## Abstract

**Background:**

Placental *Plasmodium falciparum* malaria can trigger intervillositis, a local inflammatory response more strongly associated with low birthweight than placental malaria infection alone. Fetal growth (and therefore birthweight) is dependent on placental amino acid transport, which is impaired in placental malaria-associated intervillositis. Here, we tested the hypothesis that mechanistic target of rapamycin (mTOR) signaling, a pathway known to regulate amino acid transport, is inhibited in placental malaria-associated intervillositis, contributing to lower birthweight.

**Methods:**

We determined the link between intervillositis, mTOR signaling activity, and amino acid uptake in tissue biopsies from both uninfected placentas and malaria-infected placentas with and without intervillositis, and in an in vitro model using primary human trophoblast (PHT) cells.

**Results:**

We demonstrated that (1) placental mTOR activity is lower in cases of placental malaria with intervillositis, (2) placental mTOR activity is negatively correlated with the degree of inflammation, and (3) inhibition of placental mTOR activity is associated with reduced placental amino acid uptake and lower birthweight. In PHT cells, we showed that (1) inhibition of mTOR signaling is a mechanistic link between placental malaria-associated intervillositis and decreased amino acid uptake and (2) constitutive mTOR activation partially restores amino acid uptake.

**Conclusions:**

Our data support the concept that inhibition of placental mTOR signaling constitutes a mechanistic link between placental malaria-associated intervillositis and decreased amino acid uptake, which may contribute to lower birthweight. Restoring placental mTOR signaling in placental malaria may increase birthweight and improve neonatal survival, representing a new potential therapeutic approach.

**Electronic supplementary material:**

The online version of this article (doi:10.1186/s12916-016-0759-3) contains supplementary material, which is available to authorized users.

## Background

One in six infants worldwide are born with low birthweight (<2500 g), which is the main risk factor underlying 80% of neonatal deaths [1]. The World Health Organization reaffirmed reducing the prevalence of low birthweight by 30% by 2025 as a global health priority. Malaria in pregnancy is a leading cause of low birthweight and is responsible for ~900,000 low birthweight deliveries and ~200,000 infant deaths annually [2]. Despite control measures, *Plasmodium falciparum* malaria still affects about 85 million pregnancies each year [3]. Little is known about the mechanistic link between malaria in pregnancy and low birthweight.

Malaria in pregnancy can lead to placental malaria characterized by the sequestration of *P. falciparum*-infected erythrocytes in the maternal intervillous blood space of the placenta. This can trigger the recruitment and activation of maternal immune cells, resulting in a local inflammatory response termed intervillositis. Placental malaria-associated intervillositis is more strongly associated with low birthweight than placental malaria without intervillositis [4]. The underlying mechanisms linking placental malaria-associated intervillositis and decreased birthweight are unknown, which hinders the development of intervention strategies aimed at improving the birthweight of infants born to malaria-infected women. Current malaria control strategies such as insecticide-treated bed nets, intermittent preventative malaria treatment of pregnant women, and supplementation of their diet have limited efficacy at improving birthweight [5, 6]. There is a significant and urgent need for additional interventions aimed directly at improving birthweight that can complement existing malaria control strategies.

Placental nutrient transfer controls fetal nutrient availability, which is a key determinant of fetal growth (and therefore birthweight). The placental capacity to transfer nutrients is highly dependent on the expression and function of nutrient transporters in the syncytiotrophoblast (the nutrient transporting epithelium of the human placenta) [7]. System A is a group of amino acid transporters that mediate the uptake of non-essential neutral amino acids. Decreased placental System A activity has been associated with decreased birthweight both in humans [8, 9] and in animal models [10, 11]. Importantly, the magnitude of the decrease in placental System A activity correlates with the severity of fetal growth restriction in women [8] and placental System A activity is decreased before fetal growth restriction is observed in animal models [10, 11]. This suggests that downregulation of placental System A activity directly contributes to reduced birthweight. We previously demonstrated that placental malaria-associated intervillositis reduced both the expression and activity of System A transporters, and that System A activity and birthweight are positively correlated in placental malaria [12]. However, the mechanism(s) by which placental malaria-associated intervillositis impacts placental System A activity is unknown.

The mechanistic target of rapamycin (mTOR) signaling pathway is a nutrient-sensing pathway that regulates cell growth, proliferation, and metabolism in response to hormones, growth factors, and nutrient availability (Additional file 1: Table S1). It exists as two protein complexes: mTOR complex 1 (mTORC1), the master regulator of protein translation and cell growth and proliferation; and mTOR complex 2 (mTORC2), which regulates cytoskeletal organization and cellular metabolism. mTOR is expressed in the placental syncytiotrophoblast where it regulates amino acid uptake by influencing the trafficking of transporters to the plasma membrane [13]. Upstream signals such as amino acids, growth factors, free fatty acids, oxygen, and cytokines have been shown to influence placental mTOR signaling activity [14, 15]. In animal models, placental mTOR inhibition was associated with decreased placental amino acid transport [11]. Similar findings were observed in human fetal growth restriction of causes unrelated to placental malaria [16]. These data suggest that placental mTOR signaling influences fetal growth and birthweight by regulating transplacental nutrient transport in response to maternal signals.

Here, we provide for the first time evidence that inhibition of mTOR signaling is a mechanistic link between placental malaria-associated intervillositis and decreased amino acid transport, contributing to lower birthweight. Our findings open novel avenues of research to develop interventions targeting placental mTOR signaling to improve birthweight and neonatal health in malaria-exposed populations.

## Methods

Detailed materials and methods can be found in Additional file 1.

### Collection of placental samples

The College of Medicine Research Ethics Committee, University of Malawi, approved this study. Written informed consent was obtained from primiparous Malawian women. Placental villous tissue biopsies collected after delivery were grouped based on histology as described previously [4]: uninfected (no malaria, no intervillositis), placental malaria without intervillositis, and placental malaria with intervillositis. Table 1 summarizes the clinical characteristics of the study subjects.

**Table 1:** Clinical characteristics of study subjects

	Uninfected	Placental malaria without intervillositis	Placental malaria with intervillositis	*P* value
Subjects	17	7	14	
Age (years)	19	18	20	0.61
	(18–19)	(17–21)	(18–21)	
Gestational age (weeks)	40	40	40	0.87
	(38–40)	(38–40)	(38–40)	
Maternal weight at enrollment (kg)	56	56	56	0.77
	(49.5–58.5)	(52–60)	(52–56)	
Percentage of monocytes	0	2.2	8.6	0.0001
		(1.2-3)	(6.6–10.8)	
Parasitemia (%)	0	0.41	1.2	0.0001
		(0.21–0.83)	(0.66–11.2)	
Birthweight (kg)	3.0	2.8	2.9	0.54
	(2.7–3.5)	(2.6–3.1)	(2.6–3.0)	
Placental weight (g)	500	530	495	0.80
	(430–550)	(460–560)	(420–580)	
Fetal-to-placental weight ratio	5.95	5.71	5.89	0.48
	(5.2–6.48)	(4.11–6.09)	(5.26–6.20)	

Values are presented as median and interquartile range

### Primary trophoblast cell culture

Placental villous tissue samples were collected from healthy women with normal term pregnancies following written informed consent as approved by the Colorado Multiple Institutional Review Board (COMIRB-14-1073). Primary human trophoblast (PHT) cells were isolated by trypsin digestion and Percoll centrifugation as originally described [17] with modifications [18]. Syncytialization was assessed by human chorionic gonadotropin secretion. Cell viability was assessed by lactate dehydrogenase release.

### Monocyte-conditioned media

Conditioned media were prepared as previously described [12] with modifications. CD14^+^ cells were cultured in 1:1 mixture of Dulbecco’s modified Eagle’s medium (DMEM, Sigma-Aldrich) and Ham’s F-12 nutrient mixture (Invitrogen) supplemented with 10% fetal bovine serum and with penicillin/streptomycin.

### Small interfering RNA transfection

PHT cells were transfected with 10 nM DEPTOR small interfering RNA (siRNA; SASI_Hs01_00204344, Sigma-Aldrich) or with Scramble siRNA control using Lipofectamine RNAiMax transfection reagent (Thermo Scientific) according to the manufacturer’s protocol.

### Amino acid uptake

System A activity was assessed by measuring the Na^+^-dependent uptake of ^14^C-methyl-aminoisobutyric acid (MeAIB) as previously described [13].

### Western blot

Placental homogenates and PHT cell lysates were loaded and proteins separated on 4–12% Bis-Tris gels (Invitrogen) or 12% Mini-Protean protein gels (Bio-Rad) and transferred onto 0.20-μm nitrocellulose membrane (GE Healthcare) or polyvinylidene fluoride membrane (Bio-Rad). Membranes were incubated with primary antibodies: rabbit anti-4EBP-1, anti-phospho-4EBP-1 (Thr^37/46^), anti-ribosomal protein S6, anti-phospho-ribosomal protein S6 (Ser^235/236^), anti-Akt, anti-phospho-Akt (Ser^473^), and mouse anti-β-actin (Sigma-Aldrich). Horseradish peroxidase-conjugated secondary anti-rabbit and anti-mouse antibodies (Cell Signaling) were used as secondary antibodies. Proteins were visualized using chemiluminescence detection. Densitometry was performed using NIH’s ImageJ software.

### Cytokine analysis

Cytokine profiles in monocyte-conditioned media were analyzed by a multiplexed bead-based immunoassay using a panel of antibodies against human inflammatory cytokines (BD Biosciences) according to manufacturer’s instructions.

### Data presentation and statistical analysis

Data are presented as medians and scatter plots or medians and inter-quartile range. Data were analyzed and graphs designed using Prism 5 software (Graph Pad). Two-group comparisons were made using Mann–Whitney test and three-group comparisons using Kruskal–Wallis test. Spearman’s correlation test was used to assess correlations with 95% confidence interval.

## Results

### Placental mTOR signaling is inhibited in placental malaria-associated intervillositis

**Fig. 1:**
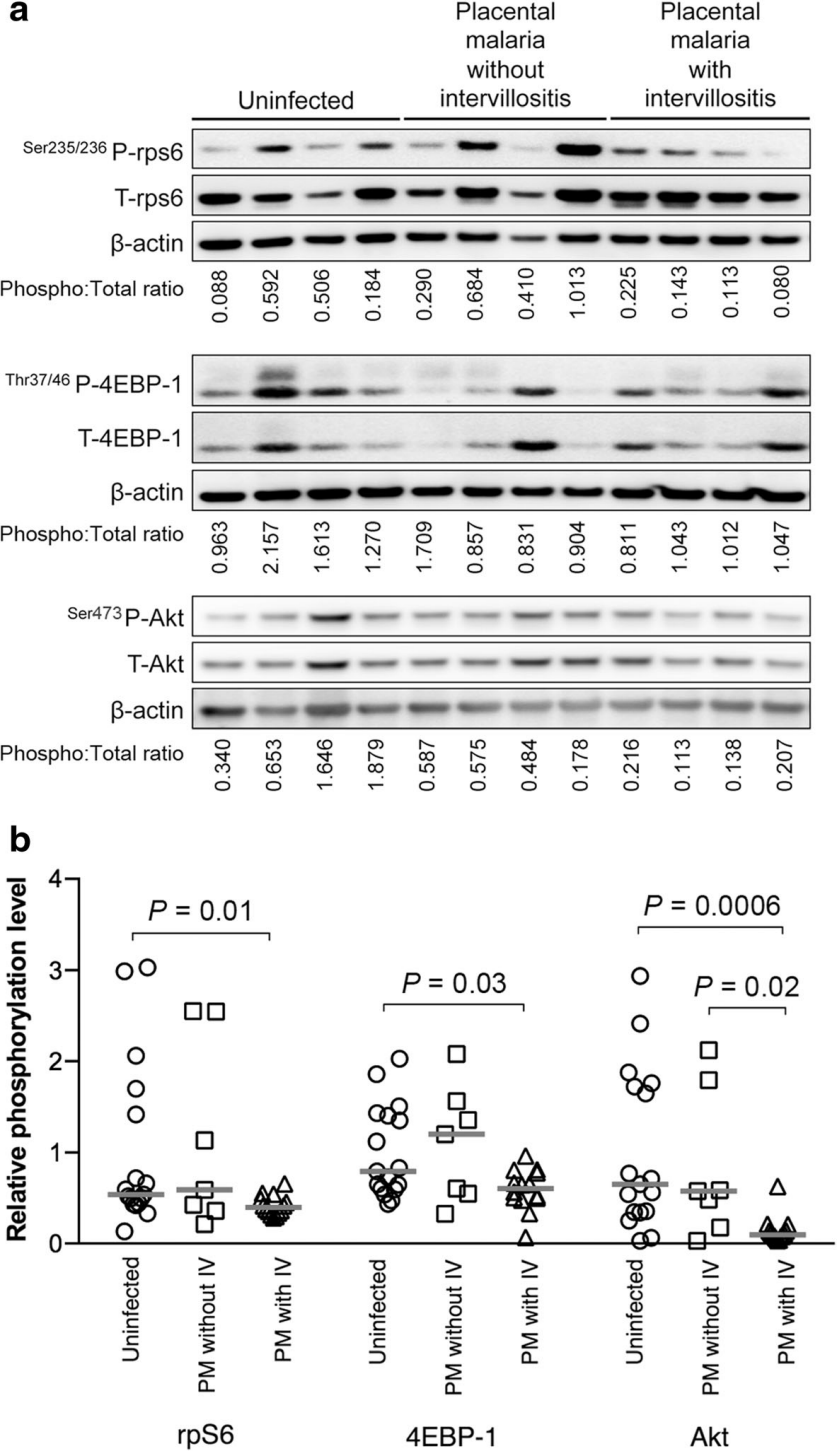
Placental malaria with intervillositis is associated with inhibition of placental mTOR signaling. mTOR activity was determined as the phosphorylated-to-total protein expression levels of rps6, 4EBP-1, and Akt in placentas grouped into uninfected (*n* = 17), placental malaria without intervillositis (*n* = 7), and placental malaria with intervillositis (*n* = 14). **a** Representative Western blot. **b** mTOR signaling activity is inhibited in placental malaria with intervillositis. *PM* placental malaria, *IV* intervillositis

We aimed to assess the impact of *P. falciparum* infection and intervillositis on mTOR signaling in human placenta using well-established mTOR functional readouts as summarized in Additional file 1: Table S1. Placental mTOR signaling activity was measured by quantifying the ratio of phosphorylated-to-total protein expression levels of downstream targets of both mTORC1 (rpS6^Ser235/236^ and 4E-BP1^Thr37/46^) and mTORC2 (Akt^Ser473^) (Fig. 1a). The activity of both mTORC1 and mTORC2 (Fig. 1b) was lower in women with placental malaria-associated intervillositis compared to uninfected placentas (rpS6: *P* = 0.01; 4EBP-1: *P* = 0.03; Akt: *P* = 0.0006) and to infected placentas without intervillositis for Akt signaling (Akt: *P* = 0.02). These results are consistent with a causal link between placental malaria-associated intervillositis and inhibition of mTOR signaling. This was further supported by the negative correlation between mTOR signaling activity and the severity of intervillositis measured as the percentage of monocytes in the intervillous space (Table 2 and Additional file 2: Figure S1).

**Table 2:** Correlation between mTOR signaling activity and percentage of monocyte, amino acid uptake and birthweight

	mTOR signaling activity^a^		
	rps6	4EBP-1	Akt
Monocytes (%)	r = −0.34	r = −0.34	r = −0.59
	(−0.60 to −0.02)	(−0.60 to −0.02)	(−0.77 to −0.32)
	*P* = 0.01	*P* = 0.01	*P* = 0.0001
Amino acid uptake (System A activity)	r = 0.28	r = 0.40	r = 0.44
	(−0.07 to 0.57)	(0.07 to 0.65)	(0.12 to 0.68)
	*P* = 0.04	*P* = 0.008	*P* = 0.004
Birthweight	r = −0.03	r = 0.27	r = 0.25
	(−0.36 to 0.30)	(−0.07 to 0.55)	(−0.08 to 0.54)
	*P* = 0.42	*P* = 0.05	*P* = 0.05

Correlation analyses are presented as r score and 95% confidence interval. These analyses include all participants (*n* = 38).

^a^Phosphorylated-to-total protein expression ratio

### Inhibition of placental mTOR signaling is associated with impaired amino acid transport and reduced birthweight

Inhibition of mTOR signaling may lead to reduced birthweight by impacting amino acid transport. We determined the relationship between placental mTOR signaling activity, amino acid transport, and birthweight. mTOR signaling showed a positive correlation with our previous microvillous plasma membrane amino acid uptake data [12] measured as System A activity (Table 2 and Additional file 2: Figure S1). This is consistent with our previous reports demonstrating that mTOR signaling is a major regulator of placental System A activity [19, 20], an important contributor to fetal growth. Importantly, we found a positive correlation between mTOR signaling activity and birthweight (Table 2 and Additional file 2: Figure S1). Birthweight was also marginally positively correlated with the degree of intervillositis (*n* = 38; r = −0.27; *P* = 0.093). Collectively, these data are consistent with the model that placental malaria-associated intervillositis decreases placental mTOR signaling, which reduces amino acid uptake and birthweight.

### Intervillositis inhibits mTOR signaling and System A amino acid transport activity in cultured primary human trophoblast cells

To establish a causal link between inhibition of mTOR signaling and reduced System A activity in placental malaria-associated intervillositis, we adapted our published protocol for studies of placental responses to placental malaria-associated intervillositis in BeWo cells for use in PHT cells [12]. Placental malaria-associated intervillositis was modeled using the conditioned medium from a monocyte/*P. falciparum*-infected erythrocyte co-culture (herein “infected conditioned medium”). This conditioned medium displayed high levels of inflammatory cytokines such as IL-1β, IL-6, IL-8, TNF, and IL-10 (Additional file 3: Figure S2). Conditioned medium from a monocyte/uninfected erythrocyte co-culture (herein “uninfected conditioned medium”) and culture medium (herein “control”) were used as controls.

**Fig. 2:**
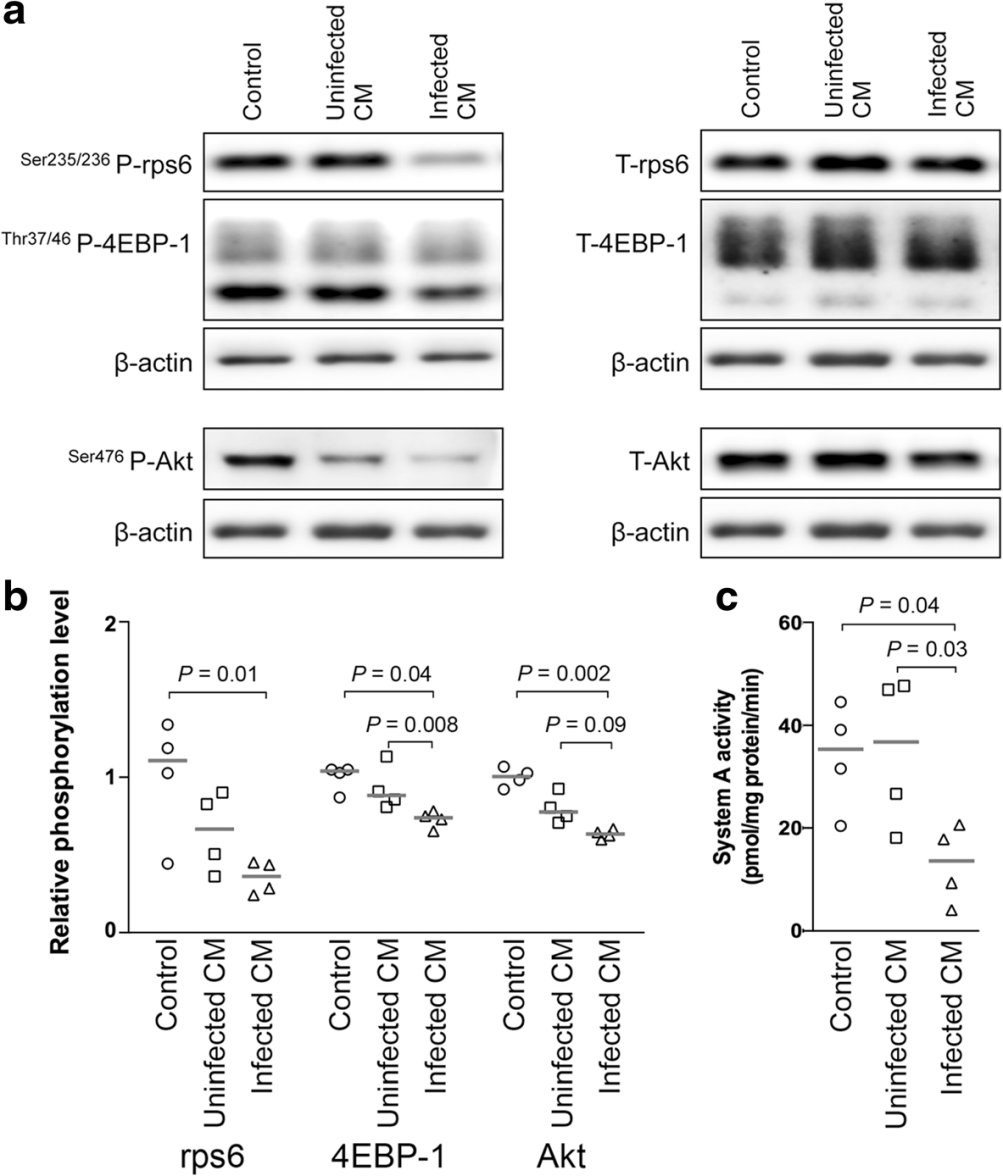
Placental mTOR signaling inhibition in an in vitro model of placental malaria-associated intervillositis. mTOR activity was determined as the phosphorylated-to-total protein expression levels of rps6, 4EBP-1, and Akt in primary human trophoblast cells exposed to infected conditioned medium, uninfected conditioned medium, and culture medium alone. **a** Representative Western blot. **b** mTOR signaling activity and **c** System A activity are decreased in primary human trophoblast cells exposed to infected conditioned medium. *n* = 4 placentas. *CM* conditioned medium, *Control* culture medium alone

Both mTORC1 and mTORC2 signaling activity were significantly lower in PHT cells exposed to infected conditioned medium compared to PHT cells exposed to uninfected conditioned medium (4EBP-1: *P* = 0.04; Akt: *P* = 0.09) or to control medium (rps6: *P* = 0.01; 4EBP-1: *P* = 0.008; Akt: *P* = 0.002) (Fig. 2). This inhibition of mTOR signaling activity was paralleled by reduced System A activity in PHT cells exposed to infected conditioned medium compared to PHT cells exposed to uninfected conditioned medium (*P* = 0.04) or to control medium (*P* = 0.03) (Fig. 2c). The viability of PHT cells was unaffected by exposure to infected conditioned media (Additional file 4: Figure S3). Collectively, these data are in agreement with our observations in women with placental malaria (Fig. 1), supporting the hypothesis placental malaria-associated intervillositis inhibits mTOR signaling, which results in reduced System A activity.

### Activation of mTOR signaling partially restores System A activity in response to placental malaria-associated intervillositis

**Fig. 3:**
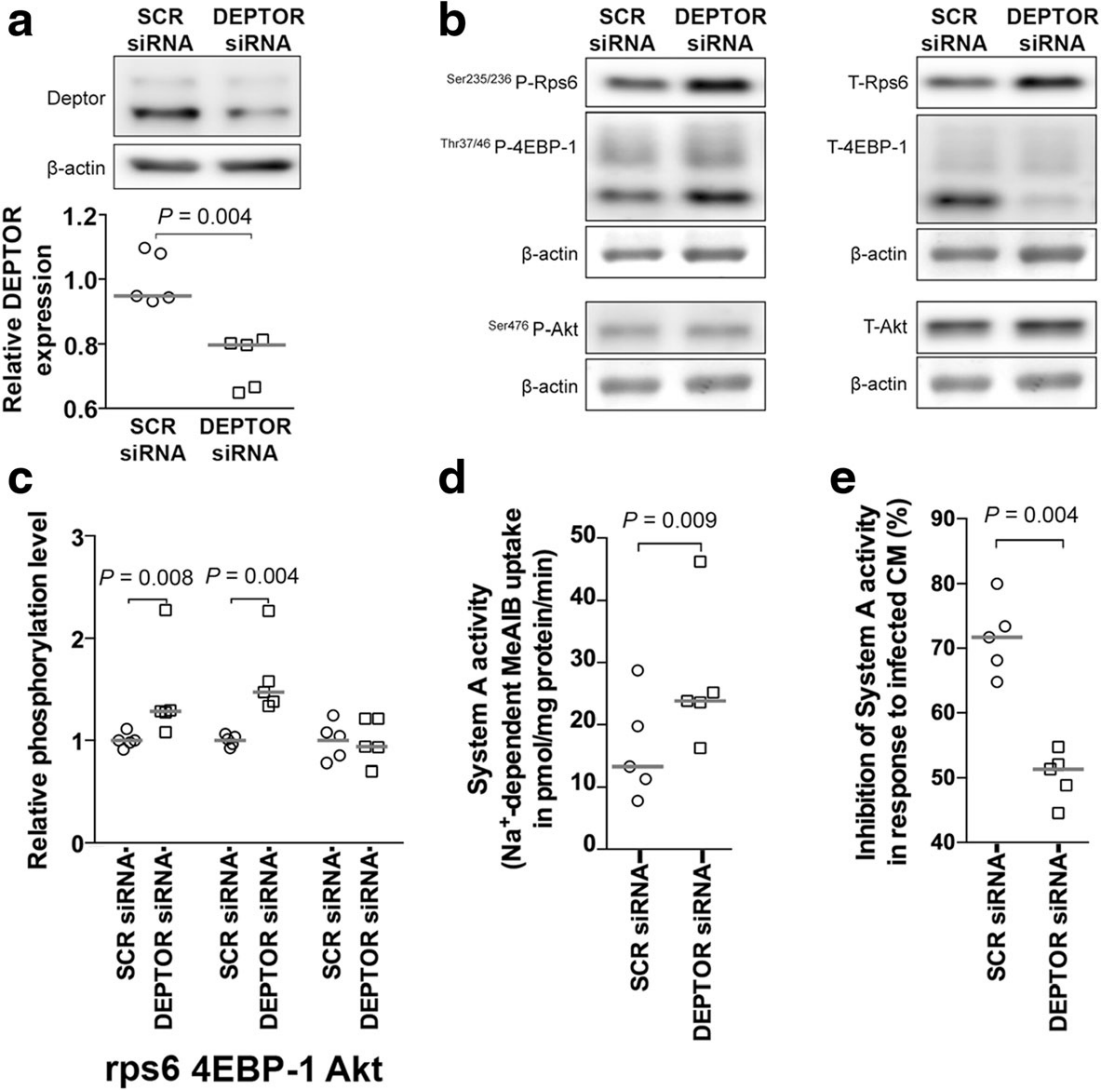
Constitutive mTOR activation by DEPTOR silencing partially restores System A activity. **a** Protein expression of DEPTOR (an endogenous mTOR inhibitor) was decreased following DEPTOR silencing in primary human trophoblast cells. mTOR activity was measured as the phosphorylated-to-total protein expression levels of rps6, 4EBP-1, and Akt in primary human trophoblast cells transfected either with DEPTOR siRNA or with a Scramble siRNA control (*SCR*). **b** Representative Western blot. DEPTOR silencing resulted in (**c**) constitutively higher mTORC1 signaling activity only and (**d**) higher System A activity. **e** DEPTOR silencing partially restored System A activity in response to infected conditioned medium. *n* = 5 placentas. *CM* conditioned medium, *SCR* scrambled

To firmly establish placental mTOR signaling inhibition as a mechanistic link between placenta malaria-associated intervillositis and reduced System A activity, we determined the effect of constitutive mTOR signaling activation on System A activity in PHT cells exposed to infected conditioned medium. To constitutively activate mTOR signaling, we silenced DEPTOR, the endogenous inhibitor of mTOR [21], using siRNA. DEPTOR silencing reduced DEPTOR protein expression by approximately 40% (*P* = 0.004) (Fig. 3a) without affecting cell differentiation and viability (Additional file 4: Figure S3). As expected, DEPTOR silencing significantly increased mTORC1 signaling activity (rps6: +44%, *P* = 0.008; 4E-BP1: +60%, *P* = 0.004; Figs 3b, c) and upregulated System A activity (+66%, *P* = 0.009; Fig. 3d) [13]. This is consistent with previous reports that silencing of DEPTOR activates mTORC1 [21].

We quantified the decrease in System A activity induced by the infected conditioned medium in PHT cells transfected with DEPTOR siRNA versus PHT cells transfected with Scramble siRNA. In cells transfected with Scramble siRNA, System A activity decreased by 72% in response to the infected conditioned medium whereas System A activity in DEPTOR-silenced cells was decreased by only 50% (Fig. 3e). This demonstrated that mTOR signaling activation by DEPTOR silencing attenuated the inhibition of System A activity in response to infected conditioned medium by approximately 30% (*P* = 0.004). This suggests that placental mTOR activation could partially restore placental amino acid uptake in response to placenta malaria-associated intervillositis.

## Discussion

Reducing the incidence of low birthweight remains a global health priority to minimize neonatal morbidity and mortality, impaired infant growth and cognitive development, and chronic diseases later in life [22]. Understanding the pathogenesis of reduced birthweight in placental malaria will provide the foundation for the development of novel interventions that complement existing malaria control approaches to directly improve fetal growth and prevent poor neonatal and pregnancy outcomes. In this study, we provide the first evidence for a role of placental mTOR signaling in decreased placental amino acid uptake and lower birthweight in placental malaria-associated intervillositis. Specifically, we demonstrate that mTOR signaling is inhibited in placental malaria-associated intervillositis and in cultured PHT cells exposed to malaria-infected conditioned media. Furthermore, we provide evidence that mTOR signaling inhibition mechanistically links intervillositis to decreased amino acid transport.

The mTOR signaling pathway has been proposed to play a central role in placental nutrient sensing [23]. This model proposes that the placenta regulates its nutrient transport function to match maternal supply and fetal demand by responding to upstream maternal signals and modulating placental function, including transplacental amino acid transport [23, 24]. In placental malaria-associated intervillositis, maternal mononuclear cells activated by infected erythrocytes in the intervillous space release inflammatory mediators that create a distinct milieu characterized by elevated levels of cytokines and chemokines such as IFN-γ, TNF, IL-10, MCP-1, MIP-1α, IL-8, CCL2, and CCL3 [25–27]. Some of these circulating inflammatory mediators could be responsible for the inhibition of placental mTOR signaling we observed in the placentas of women with placental malaria with intervillositis. The key role of intervillositis in causing inhibition of placental mTOR signaling is supported in our current study by the strong negative correlation between mTOR signaling activity and the degree of intervillositis.

Our ex vivo results reported here and in our previous publication [12] have demonstrated that placental malaria-associated intervillositis is specifically associated with impaired System A activity and that placental malaria itself (i.e., in the absence of intervillositis) is not associated with impaired System A activity. Also, we showed in our previous publication [12] that infected erythrocytes, either intact or lysed, did not impact on System A activity. These collective observations argue for a limited role (if any) of placental malaria per se on impaired System A activity. As such, we elected not to include a parasite-conditioned medium control. We attempted to model the unique milieu in the intervillous space of women with placental malaria-associated intervillositis using conditioned medium from a co-culture of monocytes and *P. falciparum*-infected erythrocytes. This conditioned medium displayed high levels of IL-1β, IL-6, IL-8, TNF, and IL-10. Cytokines such as IL-1β have been shown to inhibit mTOR activity [28] and similar cytokine profiles have been reported in malaria-infected pregnant women [26, 29]. The relevance of our in vitro model is further reinforced by the use of PHT cells, which undergo differentiation to form multinucleated syncytial islands in culture. We recapitulated the findings in women with placental malaria in this cell culture model, providing support for the concept that placental mTOR signaling inhibition mechanistically links placental malaria-associated intervillositis with decreased amino acid uptake.

mTORC1 is the master regulator of the translational machinery and activates protein synthesis by phosphorylating downstream targets including 4EBP-1 (Additional file 1: Table S1). Both in placental tissue obtained from women with placental malaria and in cultured PHT cells, we observed that placental malaria-associated intervillositis consistently inhibited phosphorylation of 4EBP-1 but not that of ribosomal protein S6 (rps6), an indirect target of mTORC1. This may be because 4EBP-1 lies directly downstream of mTORC1, unlike rps6. Reduced 4EBP-1 phosphorylation prevents protein translation initiation, decreasing protein synthesis [30]. Similar to our findings, human fetal growth restriction due to placental insufficiency unrelated to placental malaria is associated with a marked inhibition of 4EBP-1 phosphorylation [16], which could decrease protein synthesis. We speculate that inhibition of placental protein synthesis as a result of inhibition of mTOR signaling further contributes to restricted fetal growth in placental malaria-associated intervillositis.

We also observed that placental malaria-associated intervillositis decreased the phosphorylation of Akt, an mTORC2 target, to a greater extent than that of mTORC1 targets. Endoplasmic reticulum (ER) stress inhibits Akt phosphorylation [31, 32] and has been reported in the placenta of non-malaria cases of fetal growth restriction [16]. In placental malaria-associated intervillositis, the inflammatory mediators present in the intervillous space could induce syncytiotrophoblast ER stress [33], which may contribute to placental mTORC2 signaling inhibition [31] in addition to mTOR-dependent mechanisms. Given the strong association between mTORC2 activity and System A activity both ex vivo (Table 2, Additional file 2: Figure S1) and in vitro in PTH cells [20], this greater mTORC2 signaling inhibition could explain the extent of System A inhibition observed in placental malaria-associated intervillositis [12].

We established mTOR signaling as a mechanistic link between placental malaria-associated intervillositis and reduced amino acid uptake using RNAi-mediated silencing of DEPTOR, an endogenous mTOR inhibitor [21]. Knockdown of DEPTOR results in constitutive activation of mTOR signaling [34]. In the current study, DEPTOR silencing in PHT cells resulted in increased mTORC1 but not mTORC2 signaling activity. This is in agreement with Peterson and colleagues who established that knockdown of DEPTOR was sufficient to activate mTORC1 but not mTORC2 [21], causing an asymmetrical effect on mTOR signaling [35]. However, we previously observed that DEPTOR silencing in PHT cells activated both mTORC1 and mTORC2 [13]. Differences in the sequences of DEPTOR siRNA used in these studies might underlie the differing results. Given that placental malaria-associated intervillositis in the placentas of women with placental malaria and in culture PHT cells appears to inhibit mTORC2 more strongly than mTORC1, it is possible that most of the decrease in System A activity in response to placental malaria-associated intervillositis is mediated by mTORC2. The lack of significant effect of DEPTOR silencing on mTORC2 signaling could therefore explain why DEPTOR silencing only partly restored the decrease in System A activity in response to infected conditioned media. Alternatively, the partial restoration could also be due to the incomplete silencing of DEPTOR following DEPTOR siRNA transfection. Regardless, this finding suggests that increasing placental mTOR signaling could upregulate the uptake of amino acids, thereby improving birthweight.

Our results provide evidence for a causal link between placental malaria-associated intervillositis, mTOR signaling inhibition, and decreased amino acid uptake, an important determinant of fetal amino acid availability and fetal growth. The positive correlation between mTOR signaling and birthweight further supports the hypothesis that placental mTOR signaling influences fetal growth. We chose to focus on System A because this amino acid transporter is regulated by mTOR and is the placental transporter system most strongly associated with fetal growth [8]. Further work should investigate whether other placental amino acid transporters are inhibited in placental malaria-associated intervillositis. Further, a detailed profiling of inflammatory mediators in the intervillous blood and the conditioned media will help to identify factors that inhibit placental mTOR signaling.

Our findings identify placental mTOR as a potentially valuable target for interventions aimed at improving birthweight in malaria in pregnancy. There is strong interest in targeting mTOR activity in therapeutic development and various mTOR signaling regulators have recently been tested in clinical trials for other conditions [36]. Some are already used in the clinic [37, 38]. Further, recent studies provide emerging evidence that activators of mTOR signaling can be used to prevent or reverse fetal growth restriction of causes other than placental malaria [39–49]. For example, late gestation arginine treatment of women with unknown causes of fetal growth restriction increased birthweight (177 to 328 g; ~10% of normal birthweight) and decreased incidence of fetal growth restriction by 40–50% [41, 43]. Importantly, these interventions were conducted after the diagnosis of fetal growth restriction, suggesting that arginine can rescue fetal growth. Leucine treatment (in combination with other branched-chain amino acids) has also been repeatedly shown to improve fetal growth in various species and models of fetal growth restriction [47–49]. For example, leucine is essential for attenuating fetal growth restriction caused by a protein restriction in rats [47] and restores fetal weight in a mouse model of tumor-induced fetal growth restriction [49]. Significantly, these studies established that leucine supplementation also activated placental mTOR signaling. We propose that restoring placental mTOR signaling in placental malaria would enhance placental amino acid uptake and improve fetal growth and pregnancy outcomes, and should be further investigated. This could be first done using our in vitro model by testing mTOR activators for their capacity to restore amino acid uptake by PHT cells exposed to infected conditioned media. Positive hits could then be further investigated in animal models of malaria in pregnancy [50].

## Conclusions

**Fig. 4:**
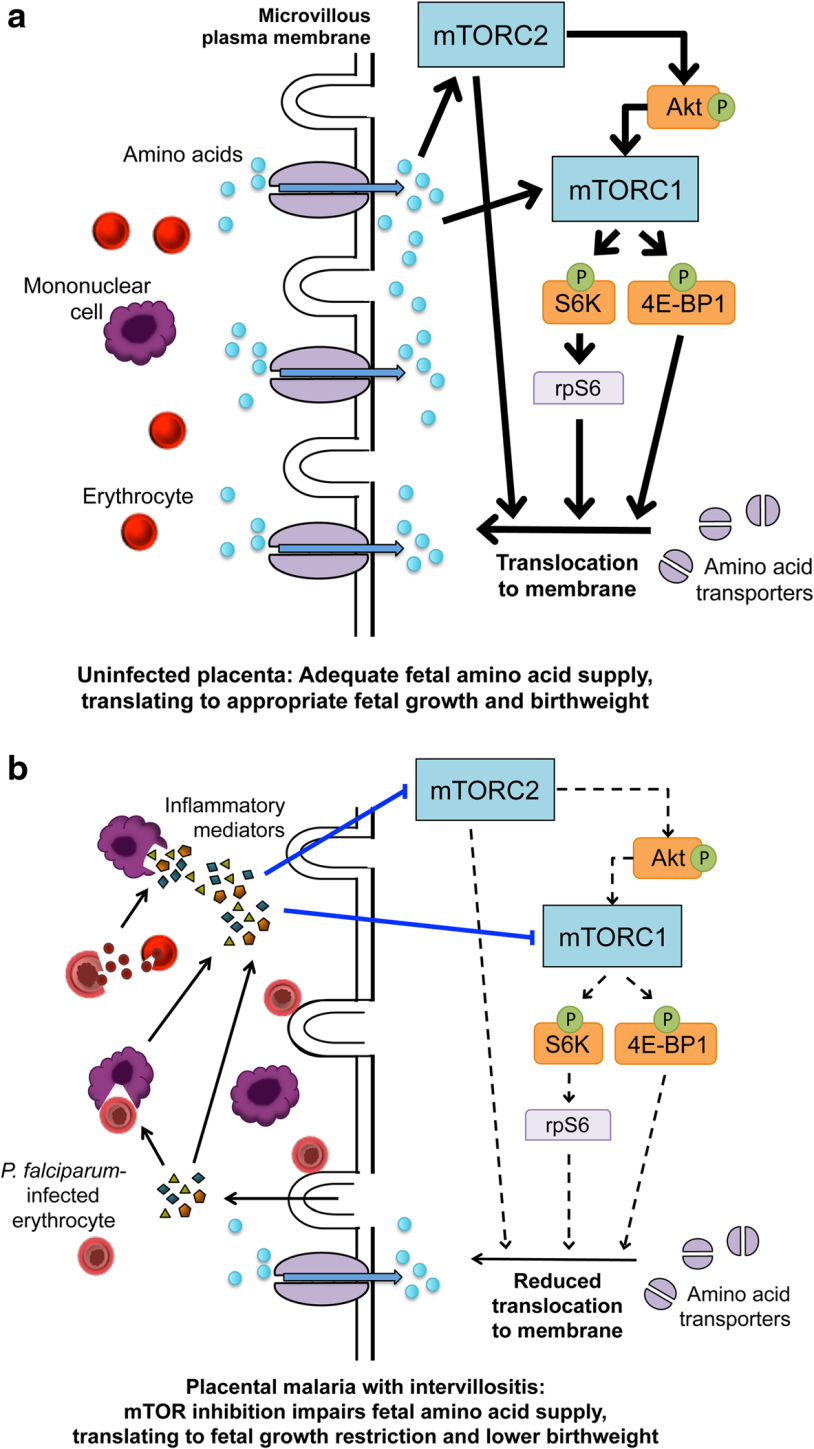
Proposed mechanism for reduced birthweight in placental malaria-associated intervillositis. **a** Under normal physiological conditions, upstream regulators of mTOR such as growth factors and hormones activate mTOR and its downstream effectors rps6, 4E-BP1, and Akt, which promote the translocation of amino acid transporters to the microvillous plasma membrane, promoting transplacental amino acid transfer and adequate fetal growth and birthweight. **b** In placental malaria-associated intervillositis, inflammatory mediators are released by monocytes after phagocytosis of *P. falciparum-*infected erythrocytes, by rupture of infected erythrocytes, and by the syncytiotrophoblast in response to the adhesion of infected erythrocytes. This inflammatory response inhibits mTOR signaling, reducing amino acid transport by decreasing the translocation of amino acid transporters to the microvillous plasma membrane. This results in a suboptimal amino acid fetal supply and reduced fetal growth and birthweight

Various malaria control measures have decreased malaria prevalence but with limited impact on the birthweight of babies born to malaria-infected women. Similarly, modest gains in birthweight have been achieved using untargeted maternal dietary supplementation [5, 6]. There is an urgent need for a better understanding of the mechanisms leading to low birthweight in malaria in pregnancy. This will allow for the development of new interventions directly aimed at improving birthweight that would be complementary to existing malaria control measures. Here, our data strongly suggest that inflammatory mediators in placental malaria-associated intervillositis inhibit placental mTOR signaling, reducing placental amino acid transport, which is likely to contribute to decreased birthweight (Fig. 4). mTOR activation increases the surface abundance of amino acid transporters in the plasma membranes of the syncytiotrophoblast allowing for increased amino acid transport [13], which may improve fetal growth and birthweight. This identifies placental mTOR as a potentially valuable target for interventions aimed at improving birthweight in malaria in pregnancy.

## Additional files

Additional file 1: Supplementary materials and methods, Table S1, and supplementary figure legends. (DOC 113 kb)

Additional file 2: Figure S1. Correlation between mTOR signaling activity and the degree of intervillositis, System A activity, and birthweight. (TIFF 491 kb)

Additional file 3: Figure S2. Cytokine profiles in conditioned media. (TIF 191 kb)

Additional file 4: Figure S3. Syncytialization and viability of cultured primary human trophoblasts. (TIF 498 kb)

## Abbreviations

4EBP-1: Eukaryotic translation initiation factor 4E-binding protein 1
Akt: Ak thymoma
CCL: CC chemokine ligand
DEPTOR: DEP domain-containing mTOR-interacting protein
IFN: Interferon
IL: Interleukin
MCP: Monocyte chemotactic protein
MIP: Macrophage inflammatory protein
mTOR: Mechanistic target of rapamycin
mTORC1: mTOR complex 1
mTORC2: mTOR complex 2
PHT: Primary human trophoblast
rpS6: Ribosomal protein 6
Ser: Serine
siRNA: small interfering RNA
Thr: Threonine
TNF: Tumor necrosis factor
