# Supplementary material for: Inhibition of placental mTOR signaling provides a link between placental malaria and reduced birthweight
Source: BMC Med. 2017 Jan 3;15:1. doi: 10.1186/s12916-016-0759-3 (PMC5209943; doi:10.1186/s12916-016-0759-3)
Supplement: Supplementary file 1 — Supplementary materials and methods, Table S1, and supplementary figure legends. (DOC 113 kb) [file 12916_2016_759_MOESM1_ESM.doc]

**Supplementary Materials and Methods**

**Collection of placental samples**

This study was approved by The College of Medicine Research Ethics Committee, University of Malawi. Written informed consent was obtained from primiparous pregnant women who delivered at term at the Queen Elizabeth Central Hospital, Blantyre, Malawi. Placental villous tissue biopsies were collected at delivery and processed as described in detail previously [12, 38]. Tissues were grouped based on placental histology: uninfected (no malaria, no intervillositis; n = 17), placental malaria without intervillositis (n = 7), and placental malaria with intervillositis (n = 14). Placental malaria was defined by the presence of infected erythrocytes in the intervillous space while intervillositis was defined as more than 5% of the intervillous cells counted being monocytes [4, 12]. Table 1 summarizes the characteristics of participants in the study. By design, the percentage of monocytes (*P* = .0001) and the placental blood parasitaemia (*P* = .0001) showed significant differences among groups.

**Cultured primary human trophoblast cells**

Placental villous tissue samples were collected from healthy women with normal term pregnancies following written informed consent as approved by the Colorado Multiple Institutional Review Board (COMIRB-14-1073). Placental tissues were coded and de-identified relevant medical information was provided through the repository. Primary human trophoblast (PHT) cells were isolated by trypsin digestion and Percoll centrifugation as originally described [17] with modifications [18, 19, 39]. Briefly, approximately 40 g villous tissue was dissected free of decidua and blood vessels, washed in phosphate-buffered saline (PBS), and digested in buffer containing HBSS, HEPES, CaCl_2_ and MgSO_4_ with trypsin (0.25%, Invitrogen) and DNAse I (Sigma-Aldrich). Cytotrophoblast cells were separated using discontinuous 10-70% Percoll gradient centrifugation. Cells between the 35% and 55% Percoll layers were collected and cultured in DMEM and Ham’s F-12 nutrient 1:1 mixture containing 10% fetal bovine serum (FBS, Atlanta Biologicals, Lawrenceville, GA), 50 μg/mL gentamicin, 60 μg/mL benzyl penicillin, and 100 μg/mL streptomycin (Sigma-Aldrich). Cells were plated in 6-well plates at a density of 2.75 × 10^6^ for subsequent protein analyses and in 12-well plates at 1.0 × 10^6^ for amino acid transport assays, and incubated in a 5% CO_2_ humidified atmosphere at 37°C. Following 18 hours of culture, attached PHT cells were washed twice in warmed Dulbecco’s PBS, and culture media were changed daily over 90 hours. Functional and expression analyses were performed at 90 hours when the cultured cytotrophoblast cells have differentiated into syncytiotrophoblast.

**Biochemical characterization and cell viability**

Trophoblast differentiation was verified by quantifying human chorionic gonadotropin (hCG) secretion into cell culture medium using an ELISA kit according to the manufacturer’s instructions (IBL-America). Viability of PHT cells was measured at 90 hours using LDH Cytotoxicity Assay kit (Thermo Scientific) according to manufacturer’s protocol. LDH release into the culture medium is an indicator of cellular toxicity.

**Generation of monocyte conditioned media**

Conditioned media were prepared as previously described [12] with modifications. Briefly, buffy coats from volunteer blood donors of the Australian Red Cross Blood Services were used to isolate CD14^+^ cells by positive immunoselection using anti-CD14 magnetic beads (Miltenyi Biotec), and purity (> 95%) was assessed by flow cytometry. *Plasmodium falciparum* (pregnancy-specific CS2 line) was maintained in O^+^ blood (Australian Red Cross Blood Services) at 2-5% haematocrit in RPMI-HEPES medium supplemented with 0.5% Albumax II (Gibco) and 25mM NaHCO_3_ at 37^0^C in 1% O_2_/5% CO_2_/94% N_2_. *P. falciparum* trophozoites were purified using magnetic cell sorting (MACS) as described elsewhere [40].

CD14^+^ cells were plated out in 24-well culture plates at a density of 5.0 × 10^5^ in 1:1 mixture of Dulbecco’s modified Eagle’s medium (DMEM, Sigma-Aldrich) and Ham’s F-12 nutrient mixture (Invitrogen) supplemented with 10% FBS and with penicillin/streptomycin, and incubated at 37^0^C in 5% CO_2_ for 1 hour to let the cells settle. Infected conditioned medium was prepared by incubating CD14^+^ cells with purified *P. falciparum* trophozoites (20 IE per CD14^+^ cell) while uninfected conditioned medium was generated by incubating uninfected erythrocytes with CD14^+^ cells (20:1). Conditioned media were collected after 24 hours of co-incubation and spun at 2,000 x*g* for 5 min to obtain cell-free supernatants.

**Small interfering RNA (siRNA) transfection and cell culture treatments**

After 18 hours of culture, PHT cells were transfected with 10nM DEPTOR siRNA (SASI_Hs01_00204344, Sigma-Aldrich) using Lipofectamine RNAiMax transfection reagent (Thermo Scientific) according to the manufacturer’s protocol. An equal concentration of non-targeting scrambled (SCR) siRNA sequence (SIC001, Sigma-Aldrich) was used as a control. siRNA and transfection agents were removed after 24 hours of incubation. After 66 hours in culture, PHT cells were treated directly with infected CM or uninfected CM supplemented with 5% FBS. At 90 hours, amino acid uptake was measured and cell lysates were collected for protein analysis.

**Amino acid uptake**

System A activity in PHT cells were assessed by measuring the Na^+^-dependent uptake of ^14^C-methyl-aminoisobutyric acid (MeAIB) as previously described [19, 20]. Briefly, cells were washed in pre-warmed Tyrode’s salt solution with Na^+^ and incubated with Tyrode’s salt solution containing ^14^C-MeAIB (20 μM at 1.19 μCi/ml) for 8 min. Cells were then lysed in distilled water. Protein content of the lysed cells was quantified using Lowry assay while the water was counted in liquid scintillation counter. Uptake activity was expressed as pmol ^14^C-MeAIB per mg protein per minute (pmol/mg protein/min).

**Western blot**

*Placental samples.* Snap-frozen biopsies of placental villous tissue were homogenized using Zirconia beads (Daintree Scientific) and proteins were extracted with radioimmunoprecipitation (RIPA) buffer containing a cocktail of protease and phosphatase inhibitors (Thermo Scientific). Extracted proteins (10 μg) were loaded and separated on pre-cast 4-12% Bis-Tris gels (Invitrogen) and transferred onto 0.20 µm nitrocellulose membrane (GE Healthcare). The membrane was blocked in 5% skim milk in Tris-buffered saline (pH 7.4) with 0.1% Tween (TBS-T) and incubated in primary antibody in TBS-T containing 1% bovine serum albumin. Primary antibodies were diluted as follows: rabbit anti-4EBP-1, anti-phospho-4EBP-1 (Thr^37/46^), anti-ribosomal protein S6, and anti-phospho-ribosomal protein S6 (Ser^235/236^), anti-Akt, anti-phospho-Akt (Ser^473^) at 1 μg/ml (Cell Signaling); and mouse anti-β-actin at 0.2 μg/ml (Sigma-Aldrich). Horseradish peroxidase (HRP)-conjugated secondary anti-rabbit (0.5 μg/ml) and anti-mouse antibody (0.2 μg/ml) (Cell Signaling) were used in TBS-T with 5% milk. Proteins were visualized by Immobilon western substrate (Millipore) according to the manufacturer’s instructions and imaged on Fujifilm LAS 3000. Densitometry was performed using NIH's ImageJ software [41]. Target protein expression was normalized to β-actin and expressed as the ratio of the signal for the phospho-specific antibody to that of the pan-reactive antibody.

*PHT cells.* Cell lysates from PHT cells were processed as described for the placental tissue homogenates with a few modifications. Extracted proteins (5 μg) were loaded and separated on pre-cast 12% Mini-Protean protein gels (Bio-Rad) and were transferred onto polyvinylidene fluoride membrane (Bio-Rad). Proteins were visualized using SuperSignal West Dura substrate (Thermo Scientific) and imaged on G:Box imaging system (Syngene).

**Cytokine analysis**

Cytokine profiles were analyzed by a multiplexed bead-based immunoassay using a panel of antibodies against human inflammatory cytokines (CBA; BD Biosciences) according to manufacturer’s instructions and acquired on a flow cytometer (BD Biosciences). The following cytokines were measured: interleukin-1β (IL-1β), interleukin-6 (IL-6), interleukin-8 (IL-8), interleukin-10 (IL-10), and tumor necrosis factor-α (TNF). Cytokine levels were log-transformed before the analysis.

**Data presentation and statistical analysis**

Data are presented in box and whiskers plot showing the median and inter-quota range. Data were analyzed and graphs designed using Prism 5 software (Graph Pad). Two-group comparisons were made using Mann-Whitney test and three-group comparisons using Kruskal-Wallis test. Spearman’s correlation test was used to assess correlations with 95% confidence interval.

**Table S1. mTOR pathway and markers assessed.** The mTOR complexes (mTORC) 1 and 2 activate various targets. * denotes targets assessed in this work. A glossary of terms used in the proposal is below.

**Figure S1. Cytokine profiles in conditioned media.** A range of inflammatory cytokines was quantified in conditioned media generated using monocytes from 3 donors. Monocytes were exposed to either *P. falciparum*-infected or uninfected erythrocytes for 24 hours. CM: conditioned medium.

**Figure S2. Syncytialization and viability of cultured primary human trophoblasts.** (**A**) Syncytialization of siRNA-transfected primary human trophoblasts was assessed by the increase in the secretion of human chorionic gonadotropin (hCG) over 90 hours in culture. (**B**) The low and similar level of lactate dehydrogenase release at 90 hours confirmed the viability of cultured PHT cells. n=5 placentas. SCR: scrambled; CM: conditioned medium.

**Figure S3. Correlation between mTOR signaling activity and the degree of intervillositis, system A activity and birthweight.**

**Table S1**

| **Acronym** | **Phosphorylation site assessed** | **Name** | **Function** |
| --- | --- | --- | --- |
| **4EBP-1** | Thr 37/46 | Eukaryotic translation initiation factor 4E-binding protein 1 | Phosphorylation results in its dissociation from eIF4E and activation of mRNA translation |
| **rps6** | Ser 235/236 | Ribosomal protein S6 | Component of the 40S ribosomal subunit thought to be involved in regulating translation |
| **mTOR** | N/A | Mechanistic target of rapamycin | Central regulator of mammalian metabolism and physiology |
| **Akt** | Ser 473 | Ak thymoma | Regulates cell survival and metabolism |
| **DEPTOR** | N/A | DEP domain-containing mTOR-interacting protein | Endogenous negative regulator of mTOR |
| **eIF4E** | N/A | Eukaryotic translation initiation factor 4E | Directs ribosomes to the cap structure of mRNAs for translation |
