# Supplementary figures and images for: Inhibition of placental mTOR signaling provides a link between placental malaria and reduced birthweight
Source: BMC Med. 2017 Jan 3;15:1. doi: 10.1186/s12916-016-0759-3 (PMC5209943; doi:10.1186/s12916-016-0759-3)

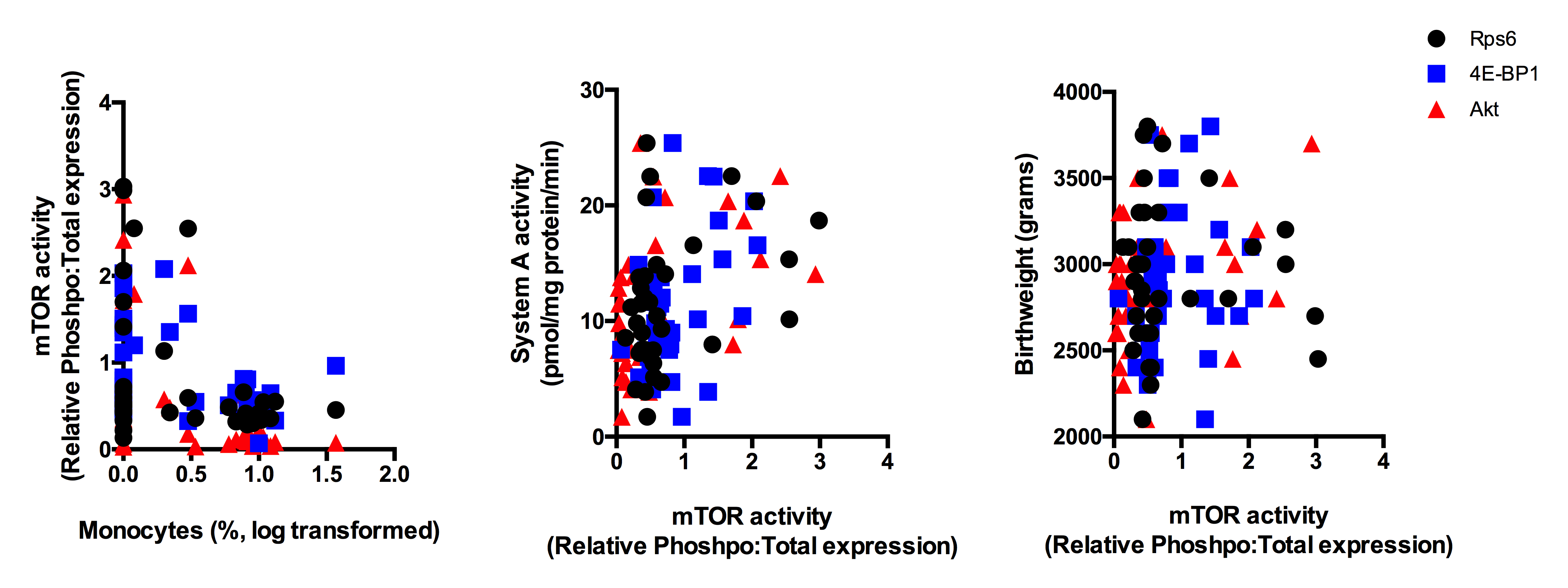

Supplement: Supplementary file 2 — Correlation between mTOR signaling activity and the degree of intervillositis, System A activity, and birthweight. (TIFF 491 kb) [file 12916_2016_759_MOESM2_ESM.tiff]

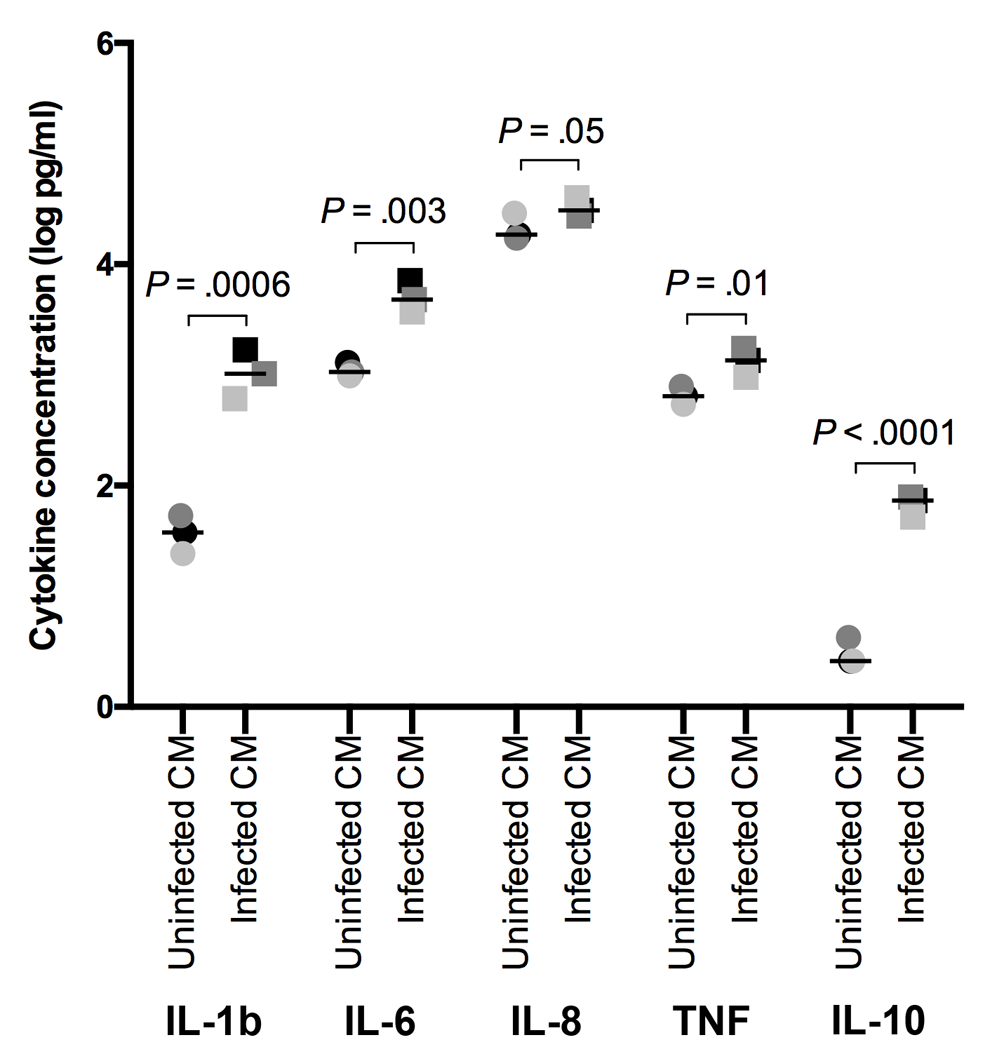

Supplement: Supplementary file 3 — Cytokine profiles in conditioned media. (TIF 191 kb) [file 12916_2016_759_MOESM3_ESM.tif]

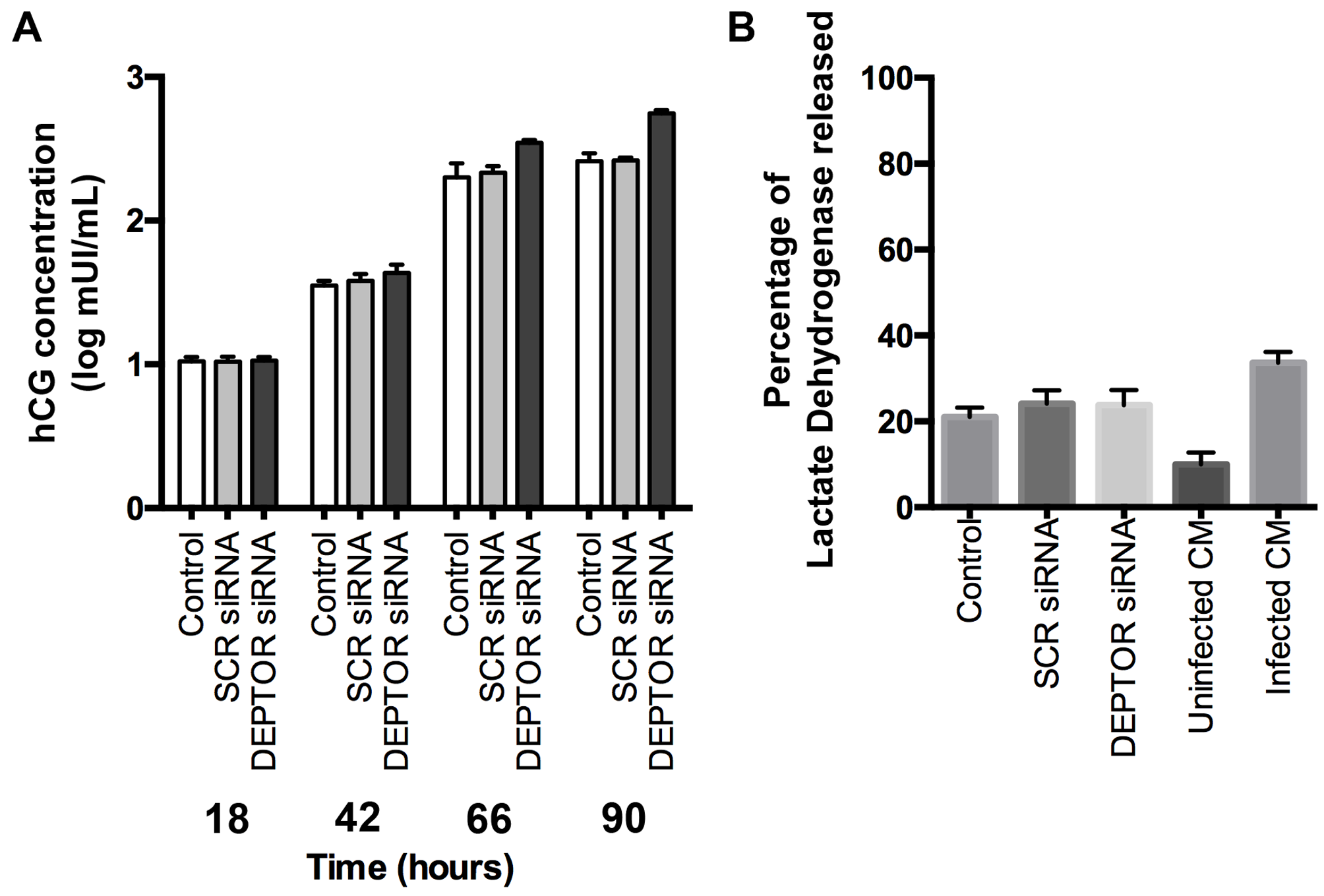

Supplement: Supplementary file 4 — Syncytialization and viability of cultured primary human trophoblasts. (TIF 498 kb) [file 12916_2016_759_MOESM4_ESM.tif]
